# Assessing quality of care in maternity services in low and middle-income countries: Development of a Maternity Patient Reported Outcome Measure

**DOI:** 10.1371/journal.pgph.0000062

**Published:** 2022-03-15

**Authors:** Fiona M. Dickinson, Barbara Madaj, Onesmus M. Muchemi, Charles Ameh

**Affiliations:** 1 Department of International Public Health, Liverpool School of Tropical Medicine, Liverpool, United Kingdom; 2 Department of International Public Health, Liverpool School of Tropical Medicine, Nairobi, Kenya; Burnet Institute, AUSTRALIA

## Abstract

Globally, low and middle-income countries bear the greatest burden of maternal and newborn mortality. To reduce these high levels, the quality of care provided needs to be improved. This study aimed to develop a patient reported outcome measure for use in maternity services in low and middle-income countries, to facilitate improvements in quality of care. Semi-structured interviews and focus groups discussions were conducted with women who had recently given birth in selected healthcare facilities in Malawi and Kenya. Transcripts of these were analysed using a thematic approach and analytic codes applied. Draft outcomes were identified from the data, which were reviewed by a group of clinical experts and developed into a working copy of the Maternity Patient Reported Outcome Measure (MPROM). A further sample of new mothers were asked to evaluate the draft MPROM during cognitive debriefing interviews, and their views used to revise it to produce the final proposed measure. Eighty-three women were interviewed, and 44 women took part in 10 focus group discussions. An array of outcomes was identified from the data which were categorised under the domains of physical and psychological symptoms, social issues, and baby-related health outcomes. The draft outcomes were configured into 79 questions with answers provided using a five-point Likert scale. Minor revisions were made following cognitive debriefing interviews with nine women, to produce the final proposed MPROM. In conjunction with women from the target population and clinical experts, this study has developed what is believed to be the first condition-specific PROM suitable for assessing care quality in maternity services in low and middle-income countries. Following further validation studies, it is anticipated that this will be a useful tool in facilitating improvements in the quality of care provided to women giving birth in healthcare facilities in these settings.

## Introduction

### Maternal & newborn health globally

It is estimated that worldwide, nearly 300,000 women die each year giving birth [1], in addition to the approximately 4.5 million babies that are either stillborn or die within the first week of life [2, 3]. Of these, most occur in low and middle-income countries and, with good care, are preventable [4]. The targets for reducing maternal and newborn deaths included in the Millennium Development Goals (MDGs) were not achieved [5]. In order to achieve the new maternal health targets proposed in the Sustainable Development Goals (SDGs), improvements in the quality of care are essential [5].

### Quality of care

Previous policies targeting improvement in population health have largely focused on increasing the availability and access to healthcare services, with the assumption that they are of sufficient quality or that the quality will improve with availability. This, however, may not always be correct, and in some situations, patients avoid healthcare facilities with good justification [6].

Defining quality of care (QoC) has been highlighted by Donabedian [7] as a key challenge in addressing and improving it, and he questions whether it is the process of caring for patients or a goal or objective of the process. The framework developed by the WHO [8] divides quality of care into three aspects in line with the Donabedian model [7]: **Structure** ie. the health system; **Process**, combining the provision and experience of care as well as human and physical resources; and **Outcome**, which includes coverage of key practices, and people-centred and health outcomes. Combining the provision and receipt of care in one model highlights the relevance and importance of including the service user perspective in assessing healthcare.

Measuring care quality can facilitate the identification of instances of poor-quality care and assist in assessing the effectiveness of quality improvement activities. Various methods have been employed to measure QoC in maternal health, including direct observation, patient interviews and standards-based audits [9–11] but each comes with its own drawbacks, including the effect of conducting the research itself. Other methods such as reviewing medical records are largely reliant on the quality of the records, which in many resource-constrained settings, may be lacking [12]. An alternative measurement approach is the use of patient reported outcomes.

### Patient Reported Outcome Measures and their uses

Patient Reported Outcome Measures (PROMs) have been variously described but are essentially structured questionnaires that assess health or health-related quality of life outcomes recounted by the patient themselves. They are developed as a survey-type form, either paper-based or increasingly commonly, electronic. The data generated by PROMs has been used to standardise research outcomes, promote patient choice, allocate financial resources, and measure quality of care [13].

A 2019 literature review to identify existing PROMs suitable for assessing quality of care in maternity services, failed to locate any suitable tools [14]. Those that were available largely focussed on specific aspects of pregnancy or the postnatal period, such as hyperemesis gravidarum [15], gestational diabetes mellitus [16], obstetric and postpartum haemorrhage [17, 18] and postnatal depression [19]. The only PROM that explored pregnancy and childbirth more broadly was that developed by Symon et al [20], however this was an individualised tool, with outcomes specified by each woman. This lack of specificity was felt to be unsuitable for assessing the quality of care as the outcomes of interest at the time of completion might have little if anything to do with the care experienced whilst giving birth. Another limitation of the existing identified childbirth related PROMs was that they had all been developed in high-income countries (UK, USA, Netherlands, Poland, Australia and New Zealand), potentially limiting their applicability to LMICs, where 94% of maternal deaths occur [21]. In addition, a search of grey literature identified the pregnancy and childbirth standard set [22] recommended by the International Consortium for Health Outcomes Measurement (ICHOM). This comprised of different measures (eg Edinburgh Postnatal Depression Scale), as well as administrative data (including mortality), a patient satisfaction scale and generic PROMs. These were described as patient centred and developed by clinicians and researchers in higher income countries, making the set inappropriate for our purposes.

Subsequently, the Obstetric quality of recovery score was published (ObsQoR-11) [23], a PROM designed to assess recovery from caesarean section, and based on the previous quality-of-recovery tool for general surgery. This was subsequently revised to form the ObsQoR-10, a slightly shorter version of the initial PROM and evaluated for use with women immediately following vaginal delivery [24]. Although these tools assessed aspects of recovery following childbirth, they were largely limited to the physical domain (items included pain, nausea, dizziness, mobilisation, and ability to feed baby without assistance), with the only psychological item relating to feeling ‘in control’. The time frame for response specified by the ObsQoR-11 and 10 was the previous 24 hours and the tool was administered on day 1 of the postnatal period. As is common with other tools described above, the ObsQoR-10 was developed and validated in high income countries (USA & UK). These factors would potentially limit the applicability of the ObsQoR-10 in a low-income setting, among postnatal women following discharge from hospital. The lack of tools suitable for assessing women’s global postpartum recovery was highlighted by Sultan et al [25] and Landau [26] and indicates the need for a PROM to assess more broadly, women’s health outcomes following childbirth.

A review was carried out to determine best practice when developing new PROMs. No single guideline or method was identified but a number of key aspects were recognised in order to ensure that the resulting PROM was relevant and acceptable to the patients it was addressing. These aspects included interviews with members of the target population to inform the outcome identification and establishing face validity through the use of cognitive debriefing methods. The review also indicated roles for clinicians in the PROM development process, including the identification of domains and refining outcomes classified through the patient interview process. The findings from the review formed the basis of the methodology of this study, which aimed to develop a condition-specific PROM to assess the quality of care provided to women and newborns using maternity services in LMICs.

PROMs on their own may have a small impact on improving QoC by virtue of the Hawthorne effect [27], with staff being aware that an assessment is being conducted and therefore modifying their behaviour accordingly. However, the greatest benefit from PROMs is likely to be seen when they are used in conjunction with quality improvement (QI) activities. An example of their use might be to administer a PROM to a number of facilities in order to identify those with the poorest outcomes. These could then be targeted with QI interventions and reassessed to determine the extent to which the intervention has achieved its goal. The aim of this study was to identify health outcomes which were important to and could be reported on by women who had recently given birth. These would then be developed into a PROM, the aggregated from which would be used as an indication of the quality of care provided in the respective healthcare facilities.

## Method

This study used a generic qualitative approach employing qualitative interviews and focus group discussions, to identify health outcomes important to women who had recently given birth, which were used to develop the final proposed maternity PROM. The study was divided into three phases–outcome identification, item generation, and pre-testing. This is in line with methodology recommended by the US Food & Drug Administration [28] (S1 Fig, MPROM development process).

### Phase 1: Outcome identification

Based on the initial review [14], a set of draft domains were drawn up and presented to a group of experienced clinicians working in maternity services in LMICs, the Clinician Review Group (CRG). The CRG validated the four primary domains of physical, psychological, social and baby outcomes, identified from the first literature review. A study by Sultan et al [29] proposed 13 domains to describe outpatient recovery following childbirth in the Unites States, which also broadly fit within the physical/psychological/social domains identified.

#### Recruitment

Semi-structured topic guides were developed based on the literature review and identified domains, and face to face interviews and focus group discussions were carried out by FD, OM (Kenya) and a research assistant (Malawi), with women who had recently given birth in one of 19 purposively selected healthcare facilities in Malawi and Kenya. These are largely English speaking, low and lower-middle income countries in sub-Saharan Africa, with a wide range of socio-demographics and different ethnic and tribal groups.

As noted in the introduction, there is no guideline or method for developing new PROMs, however based on a review of PROM development literature by the study team, a target of 100 women per country was estimated to be sufficient. Participants were recruited from six-week postnatal/vaccination clinics in both government and charity run healthcare facilities, in both countries. This time frame allowed time for women to have recovered from the birth, whilst still being able to remember any health outcomes. The facilities were purposively selected to include those from rural and urban health centres and hospitals.

Participant selection was largely opportunistic with women attending a facility on the day of data collection and who met the inclusion criteria, being invited to take part. Inclusion criteria comprised having recently given birth to a live baby, attending a vaccination or postnatal care clinic in a target facility and being able to give informed consent. Initial recruitment targets were set for women who had experienced either uncomplicated vaginal, complicated vaginal, or Caesarean section births, in order to include as a broad range of potential outcomes as possible.

#### Data collection and analysis

Data collection was conducted using a combination of semi-structured in-depth interviews (IDI) and focus group discussions (FGD) during September 2017 (Malawi) and January 2018 (Kenya). Topic guides focussed on the quality of care the women had received in the healthcare facilities and the health outcomes they had experienced. Interviews allowed individual women, particularly those who had experienced difficult or complicated births, to talk more openly about personal issues, in a one-to-one setting, without fear of their confidentiality being compromised, whilst FGDs enabled a dynamic discussion between women who had had similar births, drawing out the similarities and diversities of their experiences. The three data collectors were experienced midwives and researchers, two of whom were local to the included countries. Informed consent was obtained prior to inclusion in the study and interviews and FGDs were conducted in either English or the local language, depending on the preference of the participants. Interviews and FGDs were recorded with consent and stored securely. Recordings were transcribed verbatim, and where necessary, translated by professional transcribers. FGDs and interviews formed the primary source of data for the outcomes in the final proposed MPROM.

Data were analysed using an inductive thematic method [30] a flexible method of coding data, generated from the data itself. This facilitated analysis across the entire data set, to explore recurrent themes.

### Phase 2: Item generation

In order to develop the interview and FGD data into a working PROM, a list of potential outcomes was drawn up from the themes and transcripts from Phase 1. The CRG was asked to review the draft outcomes, in order to highlight any that they thought were overlapping or that needed expanding further, and identify any additional outcomes they thought were missing. The role of the CRG was to complement the contributions of the women, ensuring that the final MPROM would be suitable for assessing QoC in a clinical setting, but they were not privileged over the views expressed by the women.

Subsequently, based on the findings of the literature review examining methods of developing PROMs, the draft outcomes were expanded to form questions that could be answered by women, using the root: ‘Since the birth of your baby have you…’. This was followed by the outcome formed into a statement, such as ‘suffered from fever with shivering’. The baby related questions were constructed in a similar way using the root: ‘Since they were born, has your baby…’. Answer options were based on a five-point Likert scale, with two positive options, a neutral and two negative options. ‘Not applicable’ was given as an additional choice where appropriate.

In addition to the outcome questions, demographic questions were added, relating to the method and location of giving birth, and two questions about their health generally, since the birth of the baby and on the day of answering the questions. At this point the question set was labelled as the ‘Draft MPROM’.

### Phase 3: Pre-testing

Phase 3 of the study entailed pilot testing the draft MPROM with a group of women. This was done using cognitive debriefing techniques [31] and helped to establish face validity of the MPROM. Cognitive debriefing involved administering the draft MPROM to a small number of women in Malawi and Kenya, recruited using the same criteria as the Phase 1 participants. They were asked to complete the draft questionnaire and to talk about why they completed the questions in the way they did, how easily they understood the questionnaire, and if there were any other issues which they thought should be added. Due to practical constraints, the draft MPROM was only available in English. The feedback from the cognitive debriefing interviews was reviewed, and any issues identified, were addressed.

### Ethical considerations

Ethical approval was obtained from: LSTM, who also sponsored the study (Research protocol 17–007); National Health Services Research Committee (Malawi) (Ref: 17/05/1806); and Kenyatta National Hospital-University of Nairobi Ethics Research Committee (Kenya) (Ref: P297/06/2017). All women participating in the study were asked to give informed consent with study information sheets and consent forms being available in the local languages, as well as English. All data collected were anonymous and stored securely, separate from the consent forms. No incentives were given to women for taking part in the study, but they were given light refreshments during the data collection process, and a small contribution towards travel costs, to reduce any further impingement on their time. Women who had experienced a stillbirth or early neonatal death were excluded from the study, as there were no resources available to offer them appropriate support. All participants were reminded that participation was entirely voluntary and that they were free to withdraw at any point, for any reason, without penalty.

## Results

### Phase 1—Outcome identification

A total of 137 women, across Malawi and Kenya, took part in 83 interviews and 10 FGDs, in Phase 1 of the study. The women were aged between 18 and 45 years, with 52.6% (n = 72) having had an uncomplicated vaginal birth, 36.5% (n = 50) having a complicated vaginal birth and 11% (n = 15) giving birth by Caesarean section. 36 of the women (26.3%) of the women reported no pregnancies prior to most recent pregnancy, 84 women (61.3%) reported 1–3 previous pregnancies, and 17 women (12.4%) reported 4+ previous pregnancies. Complications associated with vaginal births included postpartum haemorrhage, second degree perineal tears or episiotomy, retained placenta, and neonatal sepsis. The babies were aged between 1 and 16 weeks at the time of the interviews. The median was 6 weeks, as this was the age at which they were scheduled to attend for routine postnatal care and vaccinations.

The women described the quality of care experienced in terms of their interactions with members of staff within the healthcare facilities, the availability of staff and the environment of the facility itself.

### Health outcomes

A range of themes were identified under the domains of Physical, Psychological, Social and Baby outcomes (S2 Fig, Development of themes per domain).

#### Physical

Physical issues reported by the women included pain, blood loss, perineal trauma and incontinence, as well as issues relating to breasts and breastfeeding, and sexual intercourse. These symptoms were also frequently reported to affect other aspects of the women’s lives such as work, domestic chores, mental health, and their ability to care for the new baby and other family members. Pain was often associated with the normal process of pregnancy, childbirth, and the postnatal period, such as after-pains, but other sources of pain included perineal trauma, Caesarean section wounds, discomfort from engorged breasts or nipple trauma, headaches, and backache. Similarly, to pain, a certain amount of vaginal blood loss was to be expected following the birth of the baby, but when more severe, its reporting was sometimes indicative of serious complications.

#### Psychological

The emotions and feelings expressed by the women ranged from happy that they and their baby had survived the birth experience to anxiety and stress about their current situations, particularly relating to finances and other family members. Some women also described what might be considered as signs of depression including self-isolation, being ‘tired of life’, crying for no obvious reason and having difficulty sleeping, although it was beyond the remit of this study to diagnose mental illness. There seemed to be little conscious awareness of the issue of mental health surrounding childbirth or the availability of treatment, with one woman commenting that “concerns do not have medication” (FGD, Malawi). The support that was available largely revolved around family, the local community or the church.

#### Social

Although not necessarily an obvious health outcome, issues relating to women’s social well-being often seemed to impact or be impacted by, other aspects of the women’s health. Issues raised by the women related to housework, relationships with partners and other family members, work and finances, and other social activities. For many of the women, particularly in Malawi, daily chores and housework often involved heavy physical tasks, such as carrying water from the water source, washing clothes by hand, or collecting firewood. Their ability to do these and other domestic tasks was reportedly affected by their physical wellbeing following the birth. Psychological consequences from the birth sometimes affected their relationships with others and problems with relationships, particularly with partners and close family members, and were reported to affect the women’s mental wellbeing, particularly where financial hardship or extramarital affairs were involved.

#### Baby

The health of the baby was reported by the mother and largely related to feeding and stomach problems or infections. There was, however, also evidence of some misunderstanding of health matters concerning the baby, including confusion between jaundice and yellow fever, and the misconception that umbilical hernias could be caused by air in the abdomen, resulting from the umbilical cord not being tied properly at birth.

In total, 26 key themes were generated from the combined Malawi and Kenya data and towards the end of the coding process there was a large degree repetition of code allocation and no new codes being generated.

### Phase 2—Item generation

Following analysis and collation of the interview and FGD transcripts from the two countries, 112 initial potential outcomes were attached to the themes. Following further review, and discussion with the CRG, these were reduced to a total of 79 draft outcomes across the four domains, through a process of combining themes where appropriate, such as “pain” which was included in several themes. We also removed draft outcomes that were difficult to define, such as “confused”, “crying” and “sleep” in relation to depression. Care was taken to ensure that the priorities of the women were preserved and where new outcomes were added, these were defined based on the interview and FGD transcripts. In total, 40 physical, 7 psychological, 17 social, and 15 baby related outcomes were included. Once formed into 79 items, these formed the draft MPROM used for pre-testing in Phase 3. Examples of the outcome and item generation process are included in S3 Fig, Examples of outcome and item generation process.

### Phase 3—Pre-testing

Nine women were asked to complete the draft MPROM using cognitive debriefing methods, four in Malawi and five in Kenya. Completion of the MPROM took approximately 20 minutes. Participants were recruited using the same criteria with the addition of being confident reading and understanding English, from two healthcare facilities used in Phase 1. The women were aged between 19 and 37, with a range of 2 to 12 weeks since the birth of their babies. Six had uncomplicated vaginal births, one had an assisted vaginal birth and two had Caesarean sections. Key issues identified from the cognitive debriefing related to comprehension of the questions, understanding and appropriateness of the answer options, and the format of the questionnaire. All the women were happy to complete the draft MPROM and felt that it would be acceptable to women who were attending postnatal clinics. No additional outcomes were suggested by the interviewees, although a few suggestions were made relating to how the PROM could be improved.

#### Comprehension of questions

A few of the women encountered difficulty in understanding a small number of terms used in the PROM. Challenges in understanding the questions could be divided into two categories: linguistic misunderstanding and conceptual misunderstanding. Linguistic misunderstandings occurred where women did not understand specific words in English but when it was explained to them, they understood the concept and were able to discuss how they would have answered the question. Examples of this included the words ‘cope’, ‘stools’, and ‘abscess’. Conceptual misunderstandings occurred when women not only did not understand the word in English but when it was explained, had no experience or understanding of the concept itself. Two particular examples of this were ‘varicose veins’ and ‘assisted vaginal delivery’.

#### Answer options

The answer options employed for the draft MPROM were based on a review of existing PROMs and gave the women a choice from five responses, ranging from strongly positive to strongly negative with a neutral option. For some questions an additional ‘Not applicable’ response was provided, where the question might not be relevant, such as bleeding from perineal trauma or relationships with other children. The ‘Not applicable’ option seemed to cause confusion among a few of the respondents, with some using it instead of a negative response, whilst others did not use it when it would have been appropriate. Participants suggested that clearer instructions detailing exactly what each response meant, might be helpful. Alternatively, the final version of the MPROM could use filter questions to avoid needing the ‘Not applicable’ option.

#### Questionnaire format

Feedback from the women suggested that they would prefer the layout of the questionnaire to be altered, so that the instructions on how to answer the questions were closer to the questions themselves. They also indicated a preference for having a gap between responses, and using a visual analogue type scale for the two questions asking about their health generally. These could be easily integrated into the final proposed MPROM.

## Discussion

### Principal findings

Following extensive interviews and FGDs with women who had recently given birth in Malawi and Kenya, this study identified a range of health outcomes relevant to them under the domains of physical, psychological, social, and baby. These outcomes were reviewed by a group of clinical experts and then developed into a draft PROM for pre-testing. The cognitive debriefing interviews used for pre-testing found that the draft MPROM was acceptable to women, and that they would be willing to complete it whilst attending the postnatal clinic. Some suggestions for improvements were made by the women relating to a few of the terms used and the layout of the PROM.

For the purposes of the pre-testing, it was not feasible to translate the draft MPROM into the local languages of Malawi and Kenya, so it was necessary to only recruit women who were confident in the use of English.

### Strengths and limitations

The previously published systematic review [14] was not able to identify an existing PROMs suitable for assessing quality of hospital-based maternity care, making the MPROM the first of its kind. It is also the first PROM relating to pregnancy and childbirth, developed specifically for women on LMICs, a population who carry the greatest burden of maternal and newborn mortality, globally. A key strength of this study was the relatively large number of women from two LMICs, who were included in outcome identification data collection. These interviews and FGDs ensured as broad a range of experience and perspectives as possible, to maximise the applicability of the final PROM to the target population. As there is no existing ‘gold standard’ approach to developing PROMs, the study adopted the most widely accepted methods, based on the systematic review of published information (awaiting publication), from PROM development studies in other specialities, as well as the widely cited FDA recommendations [23].

Although the inclusion of women from two different countries contributed the rigour of the study, it also presented some challenges, particularly in relation to language. The two countries were chosen, partly due to the widespread use of English as an official language. However, for many of the women interviewed, this was not their native language and necessitated the use of research assistants, to conduct some of the interviews and FGDs, and professional translation of the transcripts. The women’s limited understanding of English also presented a few challenges during the cognitive debriefing interviews and highlights the need for the final MPROM to be translated and validated into the local language of the women completing it. The importance of the provision of local language versions of tools is similarly highlighted in the EORTC guidelines on PROM development [32]. Other limitations identified during the study were the lack of understanding by some of the women of particular health problems that they had not personally experienced, and potential confusion of the causes of medical conditions such as umbilical hernias underlining the importance of cognitive debriefing [25]. The two countries that were chosen for the development of the MPROM were deliberately selected as they represented both low and middle-income countries, with varied cultural and socio-economic populations, however, further validation studies would need to be conducted in other countries in which it was applied.

### Implications and future research

It is anticipated that once validated, the MPROM would be routinely administered to women attending six-week postnatal clinics in healthcare facilities, with the aggregated findings being used at facility or district regional level to identify hospitals that would benefit from quality improvement (QI) activities. It could also be used as part of a ‘before and after’ model to assess the efficacy of QI activities. It is anticipated that further engagement with the national and regional ministries of health and other stakeholders in each country, will facilitate the utilisation and enhance the ultimate benefits of the MPROM.

To achieve widespread use of the MPROM, further research will be required to develop a scoring system, with each domain being scored separately. This could potentially enable its use without the ‘baby’ questions, for women who have experienced stillbirths or neonatal loss, although further validation research would be required as these women were not included in the initial MPROM development. Additional data collection using the tool will also ensure its validity and demonstrate that it does measure what it is expected to.

## Conclusion

This study conducted interviews and FGDs in Malawi and Kenya, with women who had recently given birth, in order to identify relevant health outcomes that they may have experienced. Outcomes were grouped under four domains, physical, psychological, social and baby. Following consultation with a group of clinical experts, the outcomes were used to develop a draft version of the MPROM, which was pre-tested with small groups of women, using cognitive debriefing methods, to establish face validity. This resulted in the production of the proposed MPROM, believed to be the first of its kind, for use in assessing the quality of care provided in healthcare facilities in LMICs. Further research is intended to establish the psychometric validity of the tool and promote its use in Kenya and Malawi.

## Supporting information

S1 Fig MPROM development process. (DOCX)

S2 Fig Development of themes per domain. (DOCX)

S3 Fig Examples of outcome and item generation process. (DOCX)
