# Supplementary material for: Assessing quality of care in maternity services in low and middle-income countries: Development of a Maternity Patient Reported Outcome Measure
Source: PLOS Glob Public Health. 2022 Mar 15;2(3):e0000062. doi: 10.1371/journal.pgph.0000062 (PMC10021686; doi:10.1371/journal.pgph.0000062)
Supplement: S1 Fig — (DOCX) [file pgph.0000062.s001.docx]

S1. MPROM development process
