# Supplementary material for: Assessing quality of care in maternity services in low and middle-income countries: Development of a Maternity Patient Reported Outcome Measure
Source: PLOS Glob Public Health. 2022 Mar 15;2(3):e0000062. doi: 10.1371/journal.pgph.0000062 (PMC10021686; doi:10.1371/journal.pgph.0000062)
Supplement: S2 Fig — (DOCX) [file pgph.0000062.s002.docx]

| Domains identified in the initial review | Themes from Malawi data | Themes from Kenya data | Combined themes on which outcomes and items were based |
| --- | --- | --- | --- |
| Physical | Pain, blood loss, perineum, urinary continence, breasts, legs, sex, other symptoms | Pain, perineal and vaginal trauma, blood loss, breasts and breastfeeding, food and eating. | Pain, blood loss, perineum/birth canal, incontinence, breasts and breastfeeding, legs and feet, sex, eating, CS wound. |
| Psychological | Happiness, anxiety and depression, fear. | Causes of stress, results of stress, fear and anxiety, depression. | Happiness, stress, depression, anxiety/fear. |
| Social | Family, husbands, finances and work, other social activities, visiting friends. | Housework, husbands and other family members, work, finances, school and training, other social activities. | Family, husband, housework, work, finances, school and training, visiting friends, other social activities. |
| Baby | Feeding, fever, stomach problems, other illnesses. | Feeding and stomach problems, respiratory problems, other problems. | Feeding, stomach problems, fever/infections, respiratory problems, other problems. |

S2. Development of themes per domain
