# Supplementary material for: Assessing quality of care in maternity services in low and middle-income countries: Development of a Maternity Patient Reported Outcome Measure
Source: PLOS Glob Public Health. 2022 Mar 15;2(3):e0000062. doi: 10.1371/journal.pgph.0000062 (PMC10021686; doi:10.1371/journal.pgph.0000062)
Supplement: S3 Fig — (DOCX) [file pgph.0000062.s003.docx]

| Malawi themes | Kenya themes | Combined themes | Draft outcomes | Draft MPROM items  Question root: Since the birth of your baby have you: |
| --- | --- | --- | --- | --- |
| Urinary continence | Urine and stools | Incontinence | Incontinence, pain, burning sensation, fistula, constipation, diarrhoea | - Found yourself passing urine before you were able to reach the toilet? - Leaked urine when you cough or laugh? - Found yourself passing urine constantly or leaking very frequently? - Had pain when you pass urine? - Had a feeling of burning when you pass urine? - Had any pain passing stools? - Had any constipation? - Had any diarrhoea? - Found yourself not being able to control when you pass stools? |
| Breasts | Breasts and breastfeeding | Breasts and breastfeeding | Pain, cracked nipples, itching, rash, abscess, swelling, hard, lack of milk supply | - Had any very bad pain in your breasts? - Had cracked or bleeding nipples? - Had any itching or a rash on your breasts? - Had an abscess on your breasts? - Had a problem with lack of milk supply? |
| Fear | Fear and anxiety | Anxiety/fear | Worried, crying, anxious, fear, afraid | - Felt very anxious or worried? - Felt fearful? |
| Anxiety and depression | Depression | Depression | Sad, confused, sleep, suicidal, tired of life, isolated, self-harm | - Felt depressed? - Felt so sad that you've been crying? - Felt suicidal or wanted to harm yourself? - Felt happy, looking forward to things? - Felt able to cope with your daily life? |
| Fever (baby) | Other problems | Fever/infections | Rash, raised temperature, eye discharge | - Had a fever? - Had a skin rash anywhere on its body? - Had red or sticky eyes? |

S3 appendix: Examples of outcome and item generation process
